# The time course of protecting a visual memory representation from perceptual interference

**DOI:** 10.3389/fnhum.2014.01053

**Published:** 2015-01-13

**Authors:** Dirk van Moorselaar, Eren Gunseli, Jan Theeuwes, Christian N. L. Olivers

**Affiliations:** Department of Cognitive Psychology, VU UniversityAmsterdam, Netherlands

**Keywords:** visual working memory, attention, protection, retro-cue, time course

## Abstract

Cueing a remembered item during the delay of a visual memory task leads to enhanced recall of the cued item compared to when an item is not cued. This cueing benefit has been proposed to reflect attention within visual memory being shifted from a distributed mode to a focused mode, thus protecting the cued item against perceptual interference. Here we investigated the dynamics of building up this mnemonic protection against visual interference by systematically varying the stimulus onset asynchrony (SOA) between cue onset and a subsequent visual mask in an orientation memory task. Experiment 1 showed that a cue counteracted the deteriorating effect of pattern masks. Experiment 2 demonstrated that building up this protection is a continuous process that is completed in approximately half a second after cue onset. The similarities between shifting attention in perceptual and remembered space are discussed.

## Introduction

Visual working memory and visual attention are separate constructs, but there is a functional overlap in their mechanisms (Awh and Jonides, 2001; Awh et al., 2006). A recent line of research involving spatial cues during the delay of visual memory tasks has demonstrated that focused attention can operate on mnemonic representations during maintenance, such that memory performance improves for cued items (Griffin and Nobre, 2003; Landman et al., 2003; Makovski and Jiang, 2007; Makovski et al., 2008; Sligte et al., 2008; Delvenne et al., 2010; Berryhill et al., 2012). The sensitivity of visual memory to attentional cues indicates that the mnemonic representations are not static, but that they can be modulated by top-down selective mechanisms.

Different mechanisms have been proposed to account for the cueing benefit. As the cue is presented after stimulus offset and there is thus no perceptual representation to enhance, Matsukura et al. (2007) proposed that cue effects are generated by a selective attention mechanism that protects the cued representation from degradation processes such as passive decay and inter-item interference. Consistent with such a protective mechanism, a cue has been found to enhance robustness to subsequent visual input from the test display (Landman et al., 2003; Makovski et al., 2008; Pertzov et al., 2013), or passively viewed images (Makovski and Jiang, 2007). It has also been argued that focused attention strengthens the binding between the content and the context of the cued representation (Kuo et al., 2011; Rerko and Oberauer, 2013). This increased binding then in turn facilitates retrieval of the content during memory test (which acts as the appropriate context).

Strengthening and protectionist mechanisms do not need to be mutually exclusive, and both types of account predict that cued items are less vulnerable to perceptual interference. The literature, however, has provided conflicting results on the effects of perceptual interference on memory performance, and how focused attention can counteract these effects. Whereas some have shown that distractions presented during maintenance impair memory performance, especially when they are of the same category as the memoranda (Dolcos et al., 2007; Zhang and Luck, 2008; Clapp et al., 2010), others have found that memory representations are insensitive to the effects of intervening masks (Irwin and Thomas, 2008; Pinto et al., 2013). Moreover, tests on the ability of a cue to counteract the effects of interference during maintenance have provided mixed results. Makovski and Jiang (2007) showed equal performance on cue trials with and without interference from passively viewed irrelevant stimuli presented between cue and test display. Pinto et al. (2013), however, found equal performance on cue trials with and without interference only when the interference was either presented in a different location (i.e., different hemifield), or contained different objects than the memoranda. In contrast, when the interference was displayed at the same location and consisted of the same objects of the to-be remembered information, performance on trials with and without interference started to diverge. Similarly mixed results have been observed with designs that incorporated interference manipulations in the interval between cue and test presentation. Whereas neither Hollingworth and Maxcey-Richard (2013) nor Rerko et al. (2014) observed a significant modulation of the cue benefit by an intervening task, Janczyk and Berryhill (2014) found a significant reduction of the cue effect when attention was shifted to another task before memory test.

The present study served two purposes. The first was to assess whether perceptual interference causes a visual memory to become less precise and/or causes it to be lost, and whether a cue can counteract such effects (Experiment 1). So far, studies that have demonstrated interference effects have shown that interference leads to an overall decline in memory performance, without specifying the nature of that decline. Second, we sought to investigate the temporal dynamics of building the cue-based protection of visual memories against perceptual interference (Experiment 2). There have been only a few studies looking at the effects of post-cue timing on memory performance, but they did not manipulate the amount of interference. Notably, Tanoue and Berryhill (2012) found that recall accuracy for cued items improved with time relative to non-cued items, with reliable benefits emerging 300 ms after the cue. Also, in a study by Pertzov et al. (2013) the cueing effect started to differentiate performance after about 300 ms. These studies show increased protection over time against interference caused by the *test* display. Such interference might be expected to occur because the test display is by definition relevant to the task, and thus the test items are processed to similar levels as the memoranda. In the present study, we are specifically interested whether cueing a memory shields it from *irrelevant* perceptual interference, and what the time course of this process is—something that cannot be assessed from these previous studies looking at the dynamics of cueing, as their designs did not include conditions of irrelevant intervening interference. The time course of protection against perceptual interference from a mask has been investigated before (Gegenfurtner and Sperling, 1993). In that study, performance on masked trials increased until it reached an asymptote at around 300 ms, suggesting that some time was required to protect the cued item against perceptual interference. However, their design did not include baseline conditions without a cue or a mask, making it difficult to assess the direct effect of the interfering mask. Moreover, as the stimuli were letters, verbal rehearsal might have aided memory performance. Finally, the experiments employed a discrete report memory test, which does not allow for a distinction between precision reduction vs. loss of memory. In the present study, we used a continuous report procedure which is a more sensitive memory measure and also allows performing a model fit to differentiate between two different aspect of maintenance, recall probability and precision of representations.

**Figure 1:**
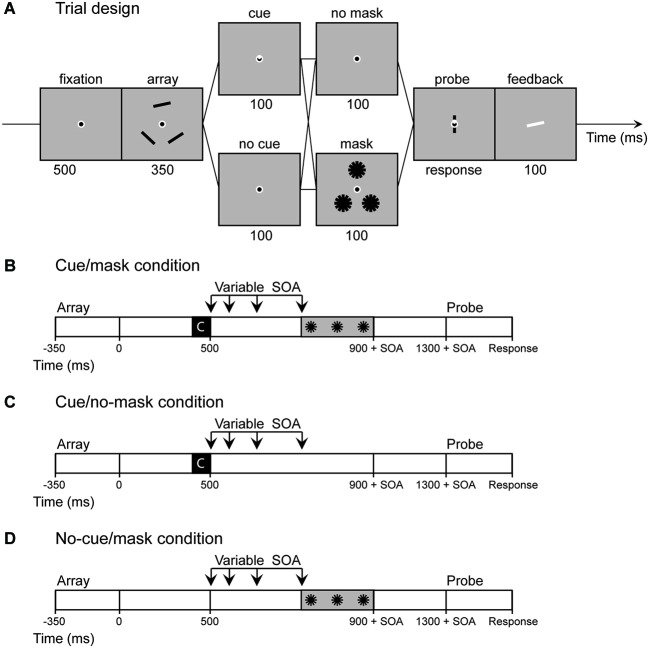
**Experiment 1: (A)** Sequence of events in a trial of Experiment 1. If a cue was present the base of a triangle inside fixation pointed towards one of the memory locations. **(B–D)** In Experiment 2 the no cue/no mask condition was omitted and the stimulus onset asynchrony (SOA) between cue display and mask display was varied (100, 200, 350, 600 ms). Note that in *cue/no-mask* condition the SOA is actually a dummy SOA as only the fixation circle was presented at the same time as the pattern masks were presented in the mask conditions. The same logic applies to the *no-cue/mask* condition, as in this condition the SOA starts at the moment of offset of the retro-cue in the cue conditions.

The basic procedure is illustrated in Figure 1. In all conditions participants were instructed to remember the orientations of three objects for a subsequent memory test. One of the memory items could then be highlighted by an endogenous cue. Furthermore between the memory and the probe display (and after the cue, if present) a pattern mask could appear. We argued that the cue would shield the memory from the mask, given sufficient time to consolidate the memory. Systematic manipulation of both cue (cue vs. no-cue) and mask (mask vs. no-mask) with variable delays then allowed us to assess at what moment in time the cue starts to have an effect and the time required for visual memory performance to stabilize upon cueing. Covertly directing attention to a location in the visual space in response to a central cue typically steadily increases between 100 and 400 ms after cue presentation (see Egeth and Yantis, 1997 for a review). A similar time-course may be observed in remembered space. Therefore, we expected performance to slowly diverge over time on masked trials with and without a cue, until performance on cued trials was indistinguishable for masked and unmasked trials.

## Experiment 1: a cue protects a memory representation against pattern masking

The present study was designed to assess the time-course of protecting the cued representation within visual memory against perceptual interference. Therefore, it was essential that the chosen measure of memory performance would be sensitive to the effects of cues over time. Recently, new methods have been developed to assess the quality of a memory representation, which are based on a continuous feature and response space instead of a binary measure (Wilken and Ma, 2004; Bays and Husain, 2008; Zhang and Luck, 2008). This method results in an estimate of the quality of recall and it has been proposed that this measure can be used to track the deployment of resources over time (Bays et al., 2011). There is some inconsistency in the literature, however, whether cues enhance only the probability of successfully recalling an item, or also its representational quality. Pertzov et al. (2013) used a continuous report task in a paradigm that required participants to recall the orientation of previously presented bars. On some trials, a probabilistic cue pointed to one of the bars. The results showed that the average angular deviation of the reported orientation from the true orientation of the target increased over time on trials without a cue, whereas performance stabilized following a valid cue. However, since they did not provide a model fit (due to an insufficient number of trials) it is unclear whether this decreased memory performance can be attributed to a decline in precision, an increase in random responses, or both. Williams et al. (2013) demonstrated that a cue leads to more precise representations as well as a lower probability of dropping the relevant information from memory (Note that these results were obtained with directed forgetting cues rather than cues to maintain an item). In contrast, Murray et al. (2013) observed enhanced probability that the target was maintained in memory following a cue, but no effect on recall precision. However, the precision calculation of Murray et al. (2013) was based on accuracy measures obtained across change detection performances in varying degrees of change. In other words, rather than reporting the feature value of the representation from a continuous scale, participants responded to a discrete set of values. Therefore, we argue that the precision estimation in Murray et al. (2013) may not have been as sensitive as continuous recall report in the studies that observed an effect of cues on precision, and this might account for the lack of such an effect (but see Souza et al., 2014a). Consequently, in Experiment 1, we used a continuous report procedure to assess whether this method would be sensitive enough to pick up differences in representational quality in the present paradigm.

In the visual domain the time course of encoding is usually studied with a masking procedure, which halts the encoding into visual memory by overwriting preceding input. It is unclear, however, whether the same procedure would be effective for studying the time course of protecting a visual memory representation, as the literature is inconclusive on the effects of masking visual memory representations. As noted before, there is evidence both in favor and against the notion that (cued) memory representations are sensitive to the effects of masking. Most of these studies, however, did not incorporate a continuous recall measure and it is thus possible that the conflicting results are due to a binary measure being only partially sensitive to the quality of representations (Awh et al., 2007). Indeed, Zhang and Luck (2008) used a continuous recall measure in a masking design and found detrimental effects of the mask. Importantly, these effects were only apparent in the probability of recall, but not in precision. Thus, Experiment 1 served to establish whether the chosen method would be sensitive enough to pick up detrimental effects of the mask, and importantly, whether such effects could be counteracted by a cue. Experiment 2 was then specifically designed to study the time-course of building this cue based protection.

Recent work has shown that increasing the proportion of invalid cue trials reduces the retro-cue benefit Gunseli et al. (in press). Similarly, using forgetting cues rather than remembering cues, Williams and Woodman (2012) found that when a cue is not 100% valid, participants may not fully focus on the cued representation. Therefore, we used 100% valid cues to ensure that participants had every reason to focus attention on the cued representation.

### Method

#### Participants

Twenty-four (13 females), aged 19–30 (*M* = 24), participated in exchange for course credit or a payment of €8 per hour. Our initial sample size contained twelve participants. However, one of the twelve participants showed a large effect completely opposite to that of the other participants As the pattern of results was consistent across the remaining participants, and since there was no objective reason to remove this outlier, we decided to test twelve more participants. All participants had normal or corrected-to-normal acuity and gave informed consent according to procedures approved by theScientific and Ethical Review Committee of the faculty of psychology and education of the VU University.

#### Apparatus, stimuli, procedure and design

A HP Compaq 8000 Elite computer running OpenSesame version 27.3 generated the stimuli on an Liyama Vision Master Pro 454 120 Hz screen and acquired the response data through the standard mouse. Participants were placed in a dimly lit room at a viewing distance of 70 cm. All stimuli were presented on a gray background (17 cd/m^2^) and a small black fixation dot (0.19°) surrounded by a white circle marked central fixation throughout each trial.

The task incorporated a cueing paradigm developed by Griffin and Nobre (2003) with a continuous response measure. Each trial started with a 500 ms fixation display. A memory display and a test display were presented sequentially, discontinued by a 1400 ms retention interval (Figure 1A). The memory display was presented for 350 ms and contained three black bars (1.62° * 0.19°) located on the corners of a triangle that subtended either 4.4° or 4.6° from fixation (memory locations switched trial by trial). The orientation of each rectangle was chosen at random with the restriction that bars within the same trial differed by at least 15°. The test display contained a randomly oriented bar at the center of fixation and a cue indicating which location was being probed. Subjects were to indicate the precise orientation of the bar at the probed location by adjusting the mouse position. After a mouse response was made, the correct orientation was indicated by a white bar for 100 ms.

In addition, we presented 100 ms cue displays, 400 ms after offset of the memory displays. If a cue was present (50% of trials), one third of the fixation dot was filled with white lines such that the base of a white triangle within fixation marked one of the memory locations (cue trials). In the other half of the trials no cue was presented such that the cue display was identical to the fixation display (*no-cue trials*). The cue displays were followed by 100-ms interfering displays presented 400 ms after offset of the cue-display (*masked trials*; 50 % of trials). These interfering displays contained three pattern masks, each comprised of 6 black bars (0°–180° in steps of 30°), identical to the memory objects, centered on the memory locations of that specific trial. Thus, in total 4 different conditions were presented (*cue/no-cue* [2] × *mask/no-mask* [2]). Trials in the *no-cue/no-mask* condition were split in half, such that the retention interval was either 900 or 1400 ms. This timing manipulation allowed us to estimate the content of visual memory both at the time the mask was presented and after the full delay.

All participants completed 48 practice trials and 8 experimental blocks of 48 trials each. Each block consisted of 12 *no-cue/no-mask*, 12 *no-cue/mask*, 12 *cue/mask* and 12 *cue/no-m*ask trials, randomly mixed, such that participants completed 96 trials in each condition. At the end of each block, feedback was given on average response error in the last block and overall in the whole experiment. Participants were encouraged to take a break between blocks.

#### Analysis

For completeness, we report a number of often-used dependent measures for each combination of participant and condition. First, for each trial, a raw measure of error was obtained by simply calculating the absolute angular deviation between the true orientation of the target and the orientation reported by the participant. The circular standard deviation (sd) of this error distribution was then calculated, which was taken as an overall measure of the quality of the memory representation (smaller values represent a better representational quality). Next, the data was fitted with a mixture model (Bays et al., 2009). This model decomposes a response distribution into three components: (1) a distribution of responses around the target, also referred to as the *probability* of recall, (2) distribution of responses around the non-target; and (3) a random response. The model also returns the concentration parameter *K* of the Von Mises distribution describing the response variability around the target and the non-target representation, which is interpreted to reflect the *precision* of the memory item. Raw error, sd, probability and precision estimates were each entered in repeated-measures ANOVAs and follow-up analyses were done with paired two-tailed* t* tests.

### Results and discussion

**Figure 2:**
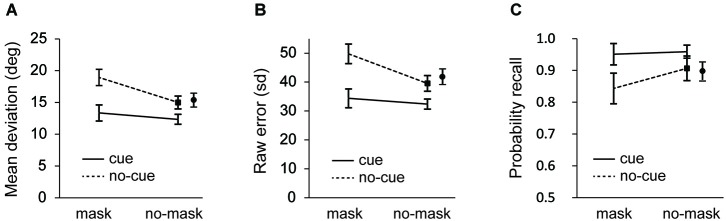
**Experiment 1: (A)** Mean average deviation **(B)** standard deviation of error distributions; and **(C)** probability of recall estimates as a function of cue and distractor-type. Data for no-cue/no-mask condition is shown separately for the short (black square) and the long interval (black circle). Error bars in all figures are condition specific, within subject 95% CI’s (Morey, 2008).

Results from Experiment 1 are displayed in Table 1 and Figure 2. Raw error scores were entered in a repeated-measures ANOVA, with within-subjects factors cue (no-cue, cue) and mask (no-mask, mask). Note, however, that the no-cue/no-mask condition consisted of a short and a long interval condition, which made a fully crossed analysis impossible, so we present the analyses for these intervals separately. In addition, as performance did not significantly differ for these two intervals, (*t*_(23)_ = 1.672, *p* = 0.11), we also present all analyses collapsed across interval.

**Table 1:** **Experiment 1: Data columns represent mean of average deviation, mean precision (standard deviation of the error in participants’ responses), mean probability of reporting the cued item, mean probability of reporting an uncued item, the mean probability of random responses, and the response variability as described by the concentration parameter *K* of the Von Mises distribution**.

Condition	Error (deg)	Precision (sd)	Pt	Pn	Pu	Recall variability (***K***)
Cue/no-mask	12 (5)	32 (11)	0.96 (0.05)	0.02 (0.04)	0.02 (0.04)	6.1 (3.6)
Cue/mask	13 (4)	34 (11)	0.95 (0.06)	0.02 (0.02)	0.03 (0.05)	5.2 (2.6)
No-cue/no-mask (short)	15 (5)	40 (13)	0.91 (0.12)	0.02 (0.03)	0.08 (0.11)	4.7 (3.0)
No-cue/no-mask (long)	16 (5)	42 (12)	0.90 (0.08)	0.04 (0.05)	0.06 (0.08)	4.8 (4.0)
No-cue/mask	19 (7)	50 (14)	0.84 (0.12)	0.03 (0.04)	0.12 (0.12)	4.0 (2.5)

*Data between brackets represents Sds*.

As seen in Figure 2A, recall performance was better after a cue (*F*_(1, 23)_ = 39.027, *p* < 0.001 for the short interval; *F*_(1, 23)_ = 49.406, *p* < 0.001 for the long interval; and *F*_(1,23)_ = 46.081, *p* < 0.001 for the collapsed data), while recall performance was impaired after presentation of the mask (*F*_(1, 23)_ = 51.283, *p* < 0.001 for the short interval; *F*_(1, 23)_ = 39.487, *p* < 0.001 for the long interval; *F*_(1,23)_ = 53.563, *p* < 0.001 for the collapsed data). Important for the present purposes, the deteriorating effect of the mask was modulated by the cue, as illustrated by a significant cue × mask interaction at the short interval (*F*_(1, 23)_ = 10.037, *p* = 0.004) and a close to significant interaction at the long interval (*F*_(1, 23)_ = 4.015, *p* = 0.057). When the data were collapsed this interaction was significant (*F*_(1,23)_ = 7.136, *p* < 0.02).

Without interference, recall performance improved following a cue relative to trials without a cue, at both long (*t*_(23)_ = 5.327, *p* < 0.001) and short intervals (*t*_(23)_ = 4.220, *p* < 0.001). The finding that mean deviation was larger in the short no-cue/no mask condition than in the cue/no-mask condition indicates that the effect of the cue cannot solely be attributed to a reduction in the duration of the retention interval. The mask also significantly affected recall performance. Without a cue, performance was worse following the mask, both at the short (*t*_(23)_ = 5.856, *p* < 0.001) and the long intervals (*t*_(23)_ = 4.337, *p* < 0.001). Importantly, as illustrated by the interaction, this deteriorating effect of the mask was modulated by the cue. Following the cue, recall performance was still impaired by the mask (*t*_(23)_ = 2.469, *p* = 0.021), but the deteriorating effect of the mask (i.e., difference in mean deviation between mask and no-mask conditions) was significantly smaller on cue than on no-cue trials. Finally, mean deviation in the cue/mask condition was smaller than in the no-cue/no-mask condition (*t*_(23)_ = 1.868, *p* = 0.075 for short interval; *t*_(23)_ = 3.136, *p* = 0.005 for long interval).

To further investigate the source of these effects, first we analyzed the overall representational quality (i.e., sd of the error distribution) across conditions. As seen in Figure 2B, recall was better following a cue (*F*_(1,23)_ = 48.440, *p* < 0.001 for the short interval; *F*_(1,23)_ = 5.437, *p* = 0.029 for the long interval; *F*_(1,23)_ = 58.394, *p* < 0.001 for the collapsed data) and it decreased after a mask (*F*_(1,23)_ = 49.970, *p* < 0.001 for the short interval; *F*_(1,23)_ = 6.911, *p* = 0.015 for the long interval; *F*_(1,23)_ = 47.147, *p* < 0.001 for the collapsed data). As was also apparent from the mean deviations, masking impaired recall performance also after a cue (*t*_(23)_ = 2.327, *p* = 0.029), but this effect of the mask was significantly smaller on cue than on no-cue trials (*F*_(1, 23)_ = 12.339, *p* = 0.002 for the short interval; (*F*_(1, 23)_ = 5.926, *p* = 0.023 for the long interval; *F*_(1,23)_ = 9.610, *p* = 0.005 for the collapsed data).

Next we fitted the mixture model. Figure 2C shows the probability of recall for the probed item (i.e., the target). Consistent with the raw error and the sd analysis, in masked trials participants showed a decreased probability of reporting the target orientation (*F*_(1, 23)_ = 5.720, *p* = 0.025 for the short interval; *F*_(1, 23)_ = 8.109, *p* = 0.009 for the long interval; *F*_(1,23)_ = 7.901, *p* = 0.01 for the collapsed data) and an increased probability of reporting the target orientation when it was cued (*F*_(1, 23)_ = 17.626, *p* < 0.001 for the short interval; *F*_(1, 23)_ = 19.674, *p* < 0.001 for the long interval; *F*_(1,23)_ = 20.229, *p* < 0.001 for the collapsed data). Importantly, these main effects were accompanied by a trend towards an interaction (*F*_(1, 23)_ = 3.284, *p* = 0.083 for the short interval; *F*_(1, 23)_ = 3.495, *p* = 0.074 for the long interval; *F*_(1,23)_ = 3.914, *p* = 0.06 for the collapsed data), reflecting a significant effect of the mask without a cue (*t*_(23)_ = 2.233, *p* = 0.036 for the short interval; *t*_(23)_ = 2.533, *p* = 0.019 for the long interval; *t*_(23)_ = 2.591, *p* = 0.016 for the collapsed data), but no such effect when the mask was preceded by a cue (*t* = 0.776, *p* = 0.45).

The cue and mask effects on the probability of recall estimates were accompanied by a modulation of random responses. Random responses decreased following a cue (*F*_(1, 23)_ = 11.414, *p* = 0.003 for the short interval; *F*_(1, 23)_ = 11.379, *p* = 0.003 for the long interval; *F*_(1,23)_ = 12.244, *p* = 0.002 for the collapsed data), and they increased following a mask (*F*_(1, 23)_ = 5.398, *p* = 0.029 for the short interval; *F*_(1, 23)_ = 8.549, *p* = 0.008 for the long interval; *F*_(1,23)_ = 8.286, *p* = 0.008 for the collapsed data). However, the interaction failed to reach significance at the short interval (*F* = 1.350, *p* = 0.26) and was close to significance at the long interval (*F*_(1,23)_ = 3.553, *p* = 0.072). Also, for the collapsed data there was no significant interaction (*F*_(1,23)_ = 2.516, *p* = 0.126). In contrast, no such effects were observed in the probability of reporting an uncued item. Although it deserves noting that at the long retention interval there was a smaller probability of misreporting the wrong item in memory following a cue, a difference close to significance (*F* = 3.973, *p* = 0.058; all other *F*’s < 1.384, all other* p*’s > 0.251).

Finally, the overall cueing benefit and the overall deteriorating effect of the mask were also apparent in the precision estimates of the model output. Cueing resulted in more precise representations (*F*_(1,23)_ = 7.610, *p* = 0.011 for the short interval; *F*_(1,23)_ = 5.437, *p* = 0.029 for the long interval; *F*_(1,23)_ = 7.261, *p* = 0.013 for the collapsed data) and masking resulted in less precise representations (*F*_(1,23)_ = 7.617, *p* = 0.011 for the short interval; *F*_(1,23)_ = 6.911, *p* = 0.015 for the long interval; *F*_(1,23)_ = 8.798, *p* < 0.01 for the collapsed data). Interestingly, the cue × mask interaction that was present in all previous analyses, was completely absent for precision (all *F*’s < 0.026, all *p*’s > 0.87), indicating that a mask impaired the precision of a memory representation to the same extent with and without a cue.

Experiment 1 was conducted to assess whether or not memory representations are sensitive to the effects of visual masking and if so whether this deterioration can be counteracted by a cue. While some have found that memory representations are insensitive to the effects of intervening masks (e.g., Pinto et al., 2013), others have found that masks do impair memory performance (e.g., Gegenfurtner and Sperling, 1993). Most work on the effects of masking, however, used binary response measures, which precludes the possibility to specify the nature of the decline. Here we observed that the deteriorating effect of the mask can be attributed to a decline in the representational quality and an increase in random responses (but see Zhang and Luck, 2008, who only found an effect on probability of recall).

Also, it was found that cues improved the representational quality of the cued item and as in Williams et al. (2013) this effect was present in both model parameters. Important for the present purpose, the deteriorating effect of the mask was also modulated by the cue. Makovski and Jiang (2007) found no difference between cue trials with and without interference. Here however, consistent with Pinto et al. (2013), a mask that is related to the memory content and presented on the memory locations impaired memory performance, but to a lesser extent than without a cue. Interestingly, this modulation by the cue was completely absent in the estimates of precision and any effect was visible only in the probability of recall.

In Experiment 2 we set out to investigate the temporal dynamics of building up this cue-based protection against perceptual interference. Therefore, in Experiment 2 we used a similar set-up as in Experiment 1 and systematically varied the stimulus onset asynchrony (SOA) between the cue and the pattern mask, to evaluate the temporal dynamics of building up this protection.

## Experiment 2: the time-course of protection

Experiment 1 showed that attentional shifts induced by cues protect visual memory representations from perceptual interference. Experiment 2 was designed to investigate the time course of building up this protection. We included three different cueing conditions, and combined them with SOA: *no-cue/mask*, *cue/mask and cue/no- mask* trials. The comparison between *no*-*cue/mask* and *cue/mask* trials allowed us to assess the point in time the cue started to have an effect, whereas the comparison between *cue/mask* and *cue/no-mask* trials allowed us to assess the time required for the cue to reach full protection of the representation. In Experiment 1, the cue-based protection was apparent in the raw data (i.e., average deviation and sd) as well as the probability of recall. In Experiment 2, however, we had to reduce the number of trials per condition as a result of including multiple SOAs. Therefore, in Experiment 2 we only analyzed the sd of the response distribution as the model output for probability was already less strong for Experiment 1, and becomes even less reliable with the smaller number of trials used here.

### Method

#### Participants

Seventeen young adults, aged 22–28 (*M* = 25), participated in exchange for course credit or a payment of €8 per hour. All had normal or corrected-to-normal acuity and gave informed consent according to procedures approved by the ethic commission of the VU University. One participant was excluded because she did not follow task instructions (i.e., used pen and paper during the experiment).

#### Apparatus, stimuli, procedure and design

The method was similar to Experiment 1 except for the following changes. Stimuli were presented on a Samsung SyncMaster 2233 120 Hz screen. The *no-cue/no-mask* condition was omitted as it was redundant for the purpose of Experiment 2. Moreover, to make the mask more effective, the 100 ms static mask was changed to a flickering mask: each mask display was presented three times for 100 ms discontinued by 50 ms fixation displays. Also, in all trials, the delay between the cue (*cue/no-cue*) and the interference displays (*mask/no-mask*) was varied systematically (Figures 1B–D). We chose four different SOAs ranging between 100 and 600 ms (100, 200, 350, 600). The SOA always referred to the interval between the onset of the cue display and the onset of the mask displays, even in conditions in which no cue or no mask was presented. This meant that the retention interval between memory offset and probe onset in all conditions varied between 1400 and 1900 ms depending on the selected SOA. Note that at the shortest SOA the mask was presented immediately after cue offset.

All participants completed 24 practice trials and 10 experimental blocks of 60 trials each. Each block consisted of 20 *cue/mask*, 20 *cue/ no-m*ask and 20 *no-cue/mask* randomly mixed trials, with equal number of trials for each delay duration (five for each SOA following the cue presentation).

### Results and discussion

**Figure 3:**
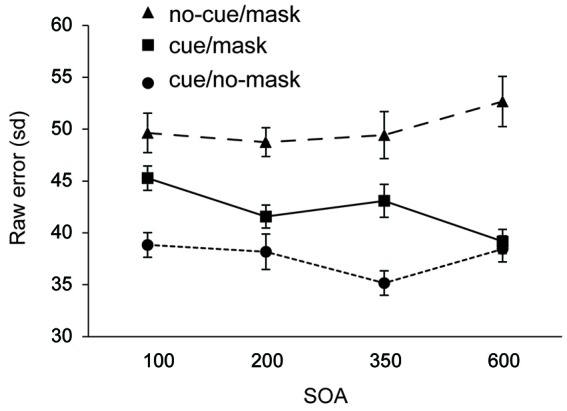
**Experiment 2: Standard deviation of error distributions for all three conditions as a function of SOA**.

Figure 3 shows the recall performance as a function of SOA and condition. For completeness, corresponding deviations are shown in Table 2. SDs and mean deviations were entered in a repeated measures ANOVA with within subjects factor SOA (100, 200, 350, 600) and condition (cue/no-mask, cue/mask, no-cue/mask). A Greenhouse-Geisser correction was applied in case of sphericity violations. There was no significant main effect for SOA (*F* = 0.68, *p* = 0.57 for sd; *F* = 1.012, *p* = 0.40 for mean deviation). There was a main effect for condition (*F*_(1.4,20.17)_ = 38.635, *p* < 0.001 for sd; *F*_(1.3,20.0)_ = 36.395, *p* < 0.001 for mean deviation), and a significant interaction (*F*_(6,90)_ = 3.287, *p* = 0.006 for sd; *F*_(6,90)_ = 3.776, *p* = 0.006 for mean deviation).

**Table 2:** **Experiment 2: Data represents mean of average deviation per condition across SOA’s**.

	SOA			
	100	250	350	600
Cue/no-mask	14.0 (3.8)	14.0 (4.8)	13.4 (3.9)	14.2 (3.5)
Cue/mask	17.1 (4.4)	16.2 (3.6)	15.9 (4.6)	14.5 (3.7)
No-cue/mask	19.6 (5.3)	18.3 (4.1)	19.8 (5.2)	21.2 (5.1)

*Data between brackets represents sd’s*.

Recall performance was lower than that observed in Experiment 1, arguably due to the more disruptive nature of the mask in Experiment 2. Across all SOA’s, recall performance was worst in the condition without a cue and with a mask (all *t*’s > 2.802, all *p*’s < 0.013 for sd; all *t*’s > 2.836, all *p*’s < 0.013 for mean deviation). Replicating findings from Experiment 1, the disruptive nature of the mask was counteracted by the cue. Results show that the cue based protection was completed within 350–600 ms following the cue, as the difference between cue/mask and cue/no-mask disappeared only at the longest SOA (*t* = 1.019, *p* = 0.32 for sd; *t* = 0.380, *p* = 0.71 for mean deviation). In contrast, at all shorter SOAs performance was significantly worse in the cue/mask condition than in the cue/no-mask condition (all *t*‘s > 2.462, all *p*’s < 0.026 for sd; all *t*‘s > 2.303, all *p*’s < 0.036 for mean deviation). Note that although cue based protection was not yet completed within the first 350 ms following the cue, the cue had a very rapid effect. Already at the 100 ms SOA performance in the cue/mask condition was better than in the no-cue/mask condition (*t* = 2.802, *p* = 0.013 for sd; *t* = 2.836, *p* = 0.013 for sd) and this effect increased over time (all *t*’s >3.038, all *p*’s < 0.008 for sd; all *t*’s > 2.913, all *p*’s < 0.011 for mean deviation). Figure 3 also suggests that incorporation of the cue slowly evolves over time. Indeed, when we analyzed the data for each condition separately, we observed a substantial linear trend (*F*_(1,15)_ = 11.544, *p* = 0.004 for sd; *F*_(1,15)_ = 12.165, *p* = 0.003 for mean deviation) for the cue/mask condition, but no such trend for the cue/no-mask baseline condition (*F* = 0.764, *p* = 0.52 for sd; *F* = 0.041, *p* = 0.84 for mean deviation) and if anything a weak trend in the opposite direction for the no-cue/mask condition (*F* = 3.45, *p* = 0.08 for sd; *F* = 3.464, *p* = 0.08 mean deviation). The observed linear trend in the cue/mask condition and no hint of such a trend in the cue/no-mask condition illustrates that the convergence of the two conditions at the longest SOA can be attributed to an increasing effectiveness of the cue over time. Thus, even though the cue had an immediate effect, optimal protection was only reached between 350 and 600 ms after cue offset.

## General discussion

Previously, it has been proposed that cueing an item in retrospect shifts attention or memory resources from a distributed mode to a focused mode and thus protects the cued item against memory degradation (Matsukura et al., 2007) or perceptual interference (Lepsien and Nobre, 2007; Makovski and Jiang, 2007). The present study was conducted to investigate the time course of implementing this protection. For this purpose a cue design was combined with a masking procedure at various SOAs. This allowed us to measure both the time required to activate the cue and the necessary time to incorporate the cue. It was found that a cue stabilizes the representational quality of the cued item such that after sufficient time it is no longer sensitive to the effects of perceptual interference. In Experiment 1 it was found that 500 ms upon cue onset the cued representation was still sensitive to the deteriorating effect of the mask, although to a lesser extent than without a cue. In Experiment 2 600 ms following cue onset there was no longer an observable effect of the cue indicating that under the present conditions it took around 500–600 ms for the cue to be fully incorporated.

The observed cue benefits add to a conflicting literature on the effects of cues on the status of memory content. Although there is ample evidence that cues improve memory performance, the source of this performance benefit remains unclear. New modeling techniques have made it possible to divide memory performance into independent measures of capacity and resolution. These models, however, have provided conflicting results. On the one hand, there is evidence that cues improve the probability of recalling the cued object, but not its precision, suggesting independence between these two measures (Murray et al., 2013; Souza et al., 2014a). On the other hand Williams et al. (2013), observed cue benefits on both the probability of recall as well as on precision. Here, consistent with Williams et al. (2013), the cue benefit was apparent in both parameters.

Retro-cue benefits have been attributed to different mechanisms, one of which is protection against perceptual interference (Makovski and Jiang, 2007) or memory degradation (Matsukura et al., 2007). The cue benefit as observed here is in line with such an account. Recall performance improved on cue trials with and without a mask, suggesting that protection also operates during maintenance (e.g., protection against decay, inter-item interference). At the same time it was found that a cue improved recall performance in the face of interference above and beyond levels if there had been no interference (cue/mask vs. no-cue/no-mask). Although this result does certainly does not rule out a protection account, it is also consistent with an active ramping up of the cued representation either because the cued item is strengthened or because the non-cued items are removed from memory (e.g., Kuo et al., 2011; Souza et al., 2014b). Here, however, we cannot dissociate between these mechanisms, which do not need to be exclusive, as with the present paradigm the effects on the uncued items in memory remain unknown.

Another important aspect of the data is the observation that without a cue the visual memory representations were sensitive to the effects of perceptual interference by a pattern mask. As noted, there is some inconsistency in the literature on the effects of masking visual memory; whereas some studies observed an effect, other studies observed equal performance in conditions with and without a mask (Dolcos et al., 2007; Irwin and Thomas, 2008; Zhang and Luck, 2008; Clapp et al., 2010; Pinto et al., 2013). In the present study the deteriorating effect of the mask was evident in a measure of precision as well as in probability of recalling the cued item. Important for the present purposes, we showed that the deteriorating effect of the mask could be counteracted by a cue. Interestingly, when applying the mixture model in Experiment 1, the only factor that appeared to contribute to this beneficial effect of the cue was an improved probability of recall, while there were no effects whatsoever on precision. This suggests that the cue protects from the mask in an all or nothing fashion: Either the item is masked and therefore lost, or it survives the mask, and then does so intact—that is, as intact as if there was no mask.

In Experiment 2 it was found that around 600 ms following a cue, a mask no longer interfered with visual memory. The time-course of this attentional shift by the endogenous cue is remarkably similar to those observed in visual working memory and in the visual attention literature. As noted in the introduction both Tanoue and Berryhill (2012) and Pertzov et al. (2013) manipulated the post-cueing time and found that it took at least about 300 ms to find a significant effect for the cue on memory performance. Similar results have also been obtained when the cue is presented concurrently with the memoranda. Bays et al. (2011) instructed participants to remember the orientation of two bars. Before the items were masked, a white disk was briefly flashed at the location of one of the memory items, either simultaneously with memory onset or 1000 ms after stimulus onset. In both situations, when the cue was valid, a significant recall advantage for the cued item developed in the first 400 ms between cue-onset and mask presentation. Moreover, it has been found that transforming a visual memory into an attentional set also takes about 400 ms to be completed (Wilschut et al., 2013). Finally, in studies that systematically varied the SOA between a predictive cue and stimulus onset, performance has been found to increased steadily until it reaches a plateau at about 400 ms (see Egeth and Yantis, 1997 for a review). Here we find a similar, though somewhat longer time course to protect a visual memory representation against perceptual interference by a pattern mask. Together these data indicate that shifting attention in both perceptual and internal space is a relatively rapid process that is characterized by a monotonic rise until an asymptote is reached and that takes at least 300 ms to be completed.

In Experiment 2, we observed significant advantages for a memory item prioritized by an endogenous retro-cue across the range of selected SOAs. That is, a recall advantage was already apparent when pattern mask was presented immediately following the offset of the cue, at 100 ms SOA. Apparently, 100 ms is already sufficient to retrieve some undamaged information from the to be tested location. Importantly, this building up of a protection at the cued location against perceptual interference continued until a maximum protection was accomplished. Experiment 2 showed that between 350 and 600 ms performance became indistinguishable from unmasked conditions. In Experiment 1, however, 500 ms following cue onset, a mask still impaired memory performance. Thus, together the data from both Experiment 1 and 2 indicate that it took around 500 to 600 ms for the cue to be optimally implemented.

In conclusion, the present results indicate that retro-cueing a visual memory item counteracts the effects of perceptual interference on memory, and in particular leads to a better representational quality. Moreover, although initial beneficial effects of the retro-cue emerge quite rapidly, protection against perceptual interference steadily evolves over time and takes between 500 and 600 ms to be completed. This time-course is similar to what has been found before for predictive cues in both mnemonic and visual selection tasks.

## Conflict of interest statement

The authors declare that the research was conducted in the absence of any commercial or financial relationships that could be construed as a potential conflict of interest.
